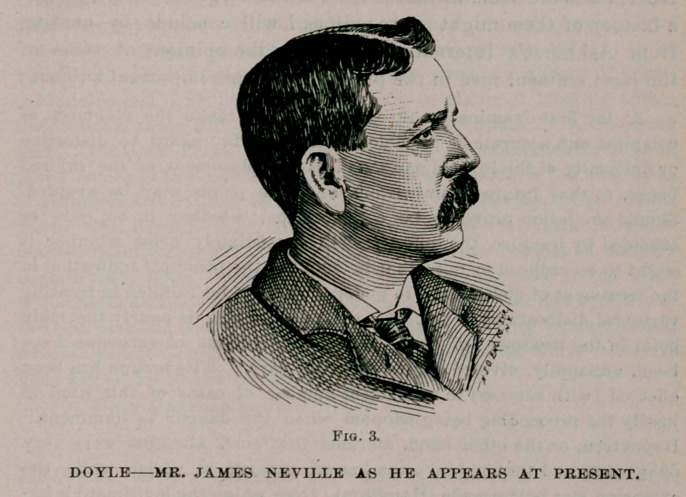# A Broken Neck—Recovery

**Published:** 1896-01

**Authors:** Gregory Doyle

**Affiliations:** Syracuse, N. Y.


### A BROKEN NECK—RECOVERY.

By GREGORY DOYLE, M. D., Syracuse, N. Y.

ON THE 18th day of October, 1894, about 6 o'clock in the evening, James Neville, of Syracuse, was thrown headlong from his wagon during a terrific runaway. He was soon after found on the roadside, apparently dead.

Physicians who were quickly summoned from the immediate neighborhood, however, detected faint signs of life, and also a deformity of the neck, which led them to suspect dislocation. An ambulance was called, and without any effort being made to relieve the deformity, he was placed in it and driven to his home, about a mile distant, the jolting over rough roads greatly aggravating his condition.

Upon his arrival home I was called, and reached his bedside about an hour after the accident. I found him unconscious and making but seven or eight respirations per minute, with a slow and intermittent pulse. His body and limbs were paralysed and his head was thrown back to an abnormal position, as correctly shown in Fig. 1. The neck was rigid and presented an unusual anterior convexity, with a deep furrow or concavity above the vertebra prominens. His general appearance certainly presented a hopeless condition.

I became satisfied, after a rapid but careful examination, that there existed a dislocation of the neck. Without further preliminaries I prepared to reduce it. Two men were told to grasp the feet and two more the head, and were directed to make careful but strong extension. At the same time I placed my right hand against the neck, just over the pomum Adami, and my left against the occiput, and while extension was being made I flexed the head forward until the chin nearly touched the breast, after which the head was returned to its normal position. This manipulation was accompanied by a clicking sensation, caused by the replacement of the dislocated vertebra. Those assisting me also felt it.

The patient immediately showed signs of relief and improved rapidly. Perceptible but feeble movements were made by all the limbs, except the right arm. He remained in a comatose condition, however, for eight or nine days, during which he had enuresis

FIG. 1.

DOYLE—MR. NEVILLE'S POSITION WHEN I ARRIVED AT HIS HOME SOON AFTER THE ACCIDENT.

with intestinal torpor. He suffered also from severe concussion of the brain, which accounts for his prolonged coma. Delirium was present for several days, during which he tossed and struggled with his attendants. On this account I did not deem it prudent to apply any retaining apparatus, such as bags of sand or bandages of any kind, but instructed the nurses to gently turn his head with the body during his frequent movements from side to side. His faithful wife, with her assistants, kept constant watch over him that no injury might occur.

His recovery was tedious, during which frequent relapses occurred. His first complaint after consciousness returned, which was on the tenth day, was of a sense of constriction about the neck, as if he were being choked. This gradually passed off, and his improvement went on without development of any serious symptoms until the present writing. He now appears to be in the best of health and able to attend to his daily avocations.

I was fully aware of the danger of lacerating the spinal cord, or perhaps an important blood-vessel, in my attempts at reduction, but, at the same time, was certain the man had but a few moments to live if not promptly relieved. Had he died during my efforts to save him I would, no doubt, have gained the credit of launching him into eternity, but as "desperate cases require desperate remedies," and in this particular case it was "do or die," I felt it my duty to afford him the only chance of life he had.

I believe many cases of broken or dislocated neck might be saved were it not for the unwarranted and culpable timidity of some surgeons. Of course, where the spinal cord is lacerated or greatly compressed in such accidents, death ensues rapidly and

FIG. 2.

DOYLE—SHOWING DISLOCATED BONES OF THE NECK, PRODUCING A BROKEN SPINAL COLUMN.

sometimes instantly. In this case, however, the compression of the spinal cord was not sufficient to cause rupture of its membranes or vessels, and the displacement of the vertebra was such as could be safely reduced by careful manipulation.

In this case the dislocation occurred at the fifth cervical vertebra. Many persons are not aware that a dislocated neck is in reality a broken neck, for the displacement of the vertebra causes a break in the spinal column, as plainly shown in Fig. 2. Therefore, a broken neck and a dislocated neck may properly be called synonymous terms.

After relieving my patient from his perilous condition, I had him removed from his couch to a bed in the next room. Several physicians who had also been summoned came in. In their estimation the case would surely prove fatal, and one especially, the senior of the convocation, declared that hemorrhage of the brain was taking place and that there was no possible chance for recovery. With this sage announcement he "stepped down and out" and strode off into outer darkness with an air of self-satisfied infallibility. His conclusions, however, were drawn from an incorrect diagnosis, as the sequel proved. I maintained at the time that the paralysis was due to the injury of the spinal cord and not to cerebral hemorrhage, and could not agree with his diagnosis

FIG. 3.

DOYLE—MR. JAMES NEVILLE AS HE APPEARS AT PRESENT.

or prognosis. I also felt justified in my efforts to relieve every unfavorable symptom that might develop, and continued unremittingly in my course until, at the end of three weeks, I had the pleasure of seeing my patient able to get on his feet and even to "take up his bed and walk" if necessary.

I have purposely delayed writing up this remarkable case until the present, in order that I might be able to record a complete recovery, and thereby sustain my diagnosis and justify the operation. Many "brilliant" operations are performed throughout the land in which every step of the proceedings is faithfully detailed in the fulsome description thereof, but curiously enough, whether through oversight or otherwise, the results are not mentioned.

Whether the patient survived or not, is the first question asked or thought of after reading of remarkable operations.

It is often trumpeted forth that Dr. Blank performed a wonderful operation which was skilfully done and was a complete success, and winds up with the meek admission that the patient died. I do not consider an operation "skilfully" done when the results are fatal, nor do I consider an operation justifiable when its performance is not imperative or the chances of relief or complete success are not of the best.

While recovery from dislocation of the neck is of rare occurrence, there are some instances recorded in surgical works, but as a history of them might prove tedious I will conclude by quoting from Ashhurst's International Surgery the opinions of some of the most eminent men in the profession on this important subject:

At the first examination the surgeon should make the diagnosis as complete and accurate as possible, particularly in regard to distortion or deformity of the injured parts and the displacement of the injured bones, so that future examinations on these points may be avoided. Should the lesion prove to be a dislocation, whether it be pure or attended by fracture, the question will immediately arise whether it ought to be reduced or not; that is, whether the principal indication in the treatment of dislocations in general ought to be fulfilled in treating vertebral dislocations or not. On this point, which is nearly the main point in the treatment of such cases, the opinions of surgeons have been, unhappily, divided. Mr. Erichsen says: "Reduction has been effected (with success) in a sufficient number of cases of this kind to justify the proceeding being adopted when the danger is imminent." Dupuytren, on the other hand, affirmed that such attempts were very dangerous, and that he had often known patients to perish while the extension was being made (Hamilton); from which the legitimate inference would follow that reduction was, in some cases, a proceeding too hazardous to be admissible. I have, however, serious doubts as to Dupuytren's assertion being well founded, for I do not find any cases whatever reported in detail, which Dupuytren could personally have known, wherein the patient perished while extension was being made. No instance of the sort is mentioned among the 394 cases that are embraced in Professor Ashhurst's tables, which clearly shows that, "in the treatment of dislocations in the cervical region, the mortality has been nearly four times greater when constitutional or general treatment has been relied on exclusively, than when attempts have been made to reduce the dislocation by extension, rotation, and the like." An inspection of the same tables also shows that in the treatment of dislocations, in the whole spinal column, "the proportion of deaths has been almost three times as large when general treatment has been exclusively used as when extension has been employed. The results of those cases which have survived have also been, as a rule, more satisfactory after extension than without it. I have already mentioned several instances in which reduction was successfully employed in the treatment of cervical dislocations, in some of which recovery would otherwise have been utterly hopeless.

It seems to me that the inference is fairly warranted, from the foregoing considerations, that extension (combined, of course, with rotation or pressure, as required,) should be employed in every case of spinal dislocation where the spinal functions are disturbed. When the diagnosis is not clear, it will be better to adopt this mode of treatment than to reject it, and I should be disposed to try it in every case where either shortening or marked angular displacement was found. (Ashhurst.) It seems to me, also, that in recent years the current of surgical opinion has, with justice, strongly set in favor of treating spinal dislocations, those with as well as those without fracture, by reducing them. Professor Porta, after carefully analysing twenty-seven cases in point, comes to the conclusion that the first indication in the treatment of vertebral dislocations, as in that of other dislocations, is to reduce them. Mr. Bryant (1878) says: "have seen several cases in which marked relief was afforded by this course, and the records of surgery contain many more. Practised with discretion, extension of the spine is doubtless a valuable means of treatment." Whenever it is applicable, the best plan of effecting reduction consists in making extension and counter extension by the gradual traction of assistants, whilst the surgeon endeavors to effect manual replacement.

307 West Genesee Street.

## Figures and Tables

**Fig. 1. f1:**
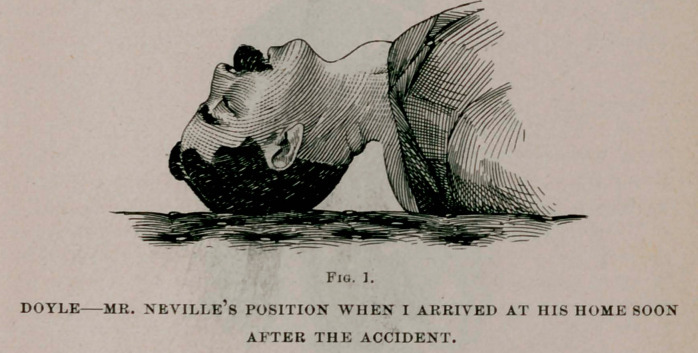


**Fig. 2. f2:**
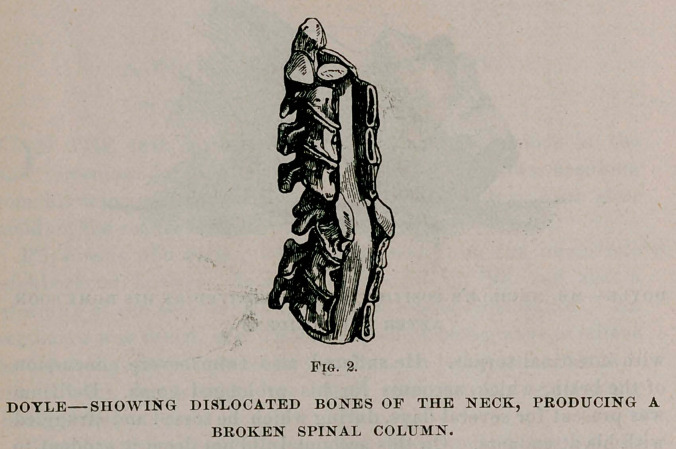


**Fig. 3. f3:**